# A Bibliometric Analysis of Publications by Anesthesia Departments in the United Arab Emirates

**DOI:** 10.7759/cureus.65878

**Published:** 2024-07-31

**Authors:** Santosh Patel

**Affiliations:** 1 Anesthesia, Tawam Hospital, Al Ain, ARE

**Keywords:** clinical researcher, country, publications, academic, anesthesia, bibliometric

## Abstract

The scholarly academic productivity and publication activities of anesthesia departments within the United Arab Emirates (UAE) are currently unknown. We undertook a bibliometric study to quantify UAE anesthetists' contributions to various categories of articles in peer-reviewed high-impact anesthesia journals. Using a PubMed-based analysis, we studied the contribution of United Arab Emirates (UAE) anesthetists towards publications in eight high-impact anesthesia journals (represented in the database for 2023 from Journal Citation Reports®, Thomson Scientific) and four anesthesia subspecialty journals over five years ranging from 2019 to 2023. Additionally, we searched each journal's website for publications related to the individual region of the UAE. We included all categories of articles except correspondences (not free-standing), meeting abstracts, and book reviews. We analyzed the following subsets comprehensively: region of the UAE, author's affiliation to the anesthesia department, publication focus, first authorship, and corresponding author status. UAE anesthetists were involved in 31 publications during the five-year study period. Over 25% of publications originated from the UAE; others were international collaborations. Overall, the anesthesia publication rate in the UAE was less than one per year for one million inhabitants. Only five hospitals contributed more than one publication. The Abu Dhabi Emirate's two main cities (Abu Dhabi and Al Ain) contributed 71% of publications. The UAE anesthetists' primary publication focus was regional anesthesia, medication error, and neurosurgical anesthesia in either the review or original articles category. Our study reveals that the current academic publication output from the anesthesia departments in the UAE is minimal. Our analysis suggests the need for increased scholarly activity, which could significantly advance anesthesia research and practice in the UAE.

## Introduction and background

Academic publications in anesthesia are a means of disseminating research findings and facilitating innovations and advancements in anesthesiology and its subspecialties. Scholars engage in research publications in anesthesia and other medical specialties for many reasons. For example, to enhance existing knowledge or forge new frontiers, disseminate their investigative findings, and propose innovative ideas [1]. In addition to contributing to the body of the literature, researchers can earn peer recognition, bolster their professional reputation, advance their careers, secure funding for future research, and influence specialty-related policy decisions [2]. Research and publications are equally vital for individual institutions, as they serve as a beacon of innovation, enhance rankings, attract academic scholars, and facilitate necessary accreditations. Moreover, countries vie to maintain or achieve a higher research ranking, reaping potential economic benefits [3].

There are a multitude of methods that are used as aids when examining national contributions to the anesthesia literature, including database searches (e.g., Medline) for articles with a department of anesthesia in the institutional affiliation field of the publication in the chosen set of journals [4]. One surrogate marker of the quality of a journal is the impact factor. Measures of scholarship within any scientific discipline should take into consideration quality and volume [2]. For anesthesia, the United States produces the most clinical research [5]. Another notable correlation for a higher research publication volume is a nation's economic strength. One study examining G-20 countries found that China and India exhibited the most publication growth, 11- and 9-fold, respectively, and are now among the top five countries regarding the number of published articles [6]. While absolute numbers country-wise are a crucial factor in ranking, it is essential to note that geographical population size varies.

Despite the economic advantage of the United Arab Emirates (UAE), its academic contribution to anesthesia publication is yet to be fully realized. Bibliometrics is the study of scholarly publications to describe publishing trends and to highlight relationships between published works based on data (e.g., authors, topics, funding) in the same way that an epidemiologist queries patient data to understand the health of a population [7]. The author aimed for a bibliometric study to quantify UAE anesthetists' contributions to different categories of articles in high-impact specialty and subspecialty anesthesia journals.

## Review

Methods

No ethics committee approval was required for this bibliographic study. We collected bibliometric data by searching PubMed to identify articles published between 2019 and 2023 in the UAE. The UAE has seven emirates. The Abu Dhabi Emirate is the largest and has three regions (Abu Dhabi, Al Ain, and Al Dhafra). We searched articles using keywords: (Region [All]) AND (Anesthesiology [ALL] or Anesthesia [ALL] or Anesthetist [AD]). We searched for individual regions. The 'region' included Abu Dhabi, Al Ain (part of the Abu Dhabi emirate), Al Dhafra (part of Abu Dhabi), Ajman, Dubai, Ras-Al Kamiah, Fujairah, Sharjah, and Uma-Al Quwain. We also thoroughly searched the individual journal's website, entering the individual region name in the search box.

We included articles from four high-impact factor anesthesia journals (represented in the 2023 database from Journal Citation Reports®, Thomson Scientific) published in North American countries (USA and Canada) and four high-impact factor journals published in Europe. In addition, we included four highest-impact factor subspecialty journals, including cardiac, neuro, obstetric, and pediatric anesthesia, to ensure a comprehensive representation of the field. 

We included all categories of articles, e.g., original research, reviews, guidelines, special articles, editorials, case reports, and correspondence (free-standing is defined as the author's own work). We excluded correspondence/letters to the editor if they were comments on the published article or the author's reply, meeting abstracts, duplicates, or articles not related to anesthesia.

We created the UAE anesthesia publication database using Microsoft Excel document software. We allocated articles to hospitals in specific regions of the UAE based on the authors' affiliation fields. We then conducted a detailed analysis of the position of UAE authors among the total number of authors mentioned in the publication and whether the UAE author was a corresponding author. We also recorded the publications for each area (region), as well as the article type and publication focus. Finally, we calculated the number of articles per million inhabitants in the UAE. The data is represented as the number or percentage of the articles.

Results

**Figure 1:**
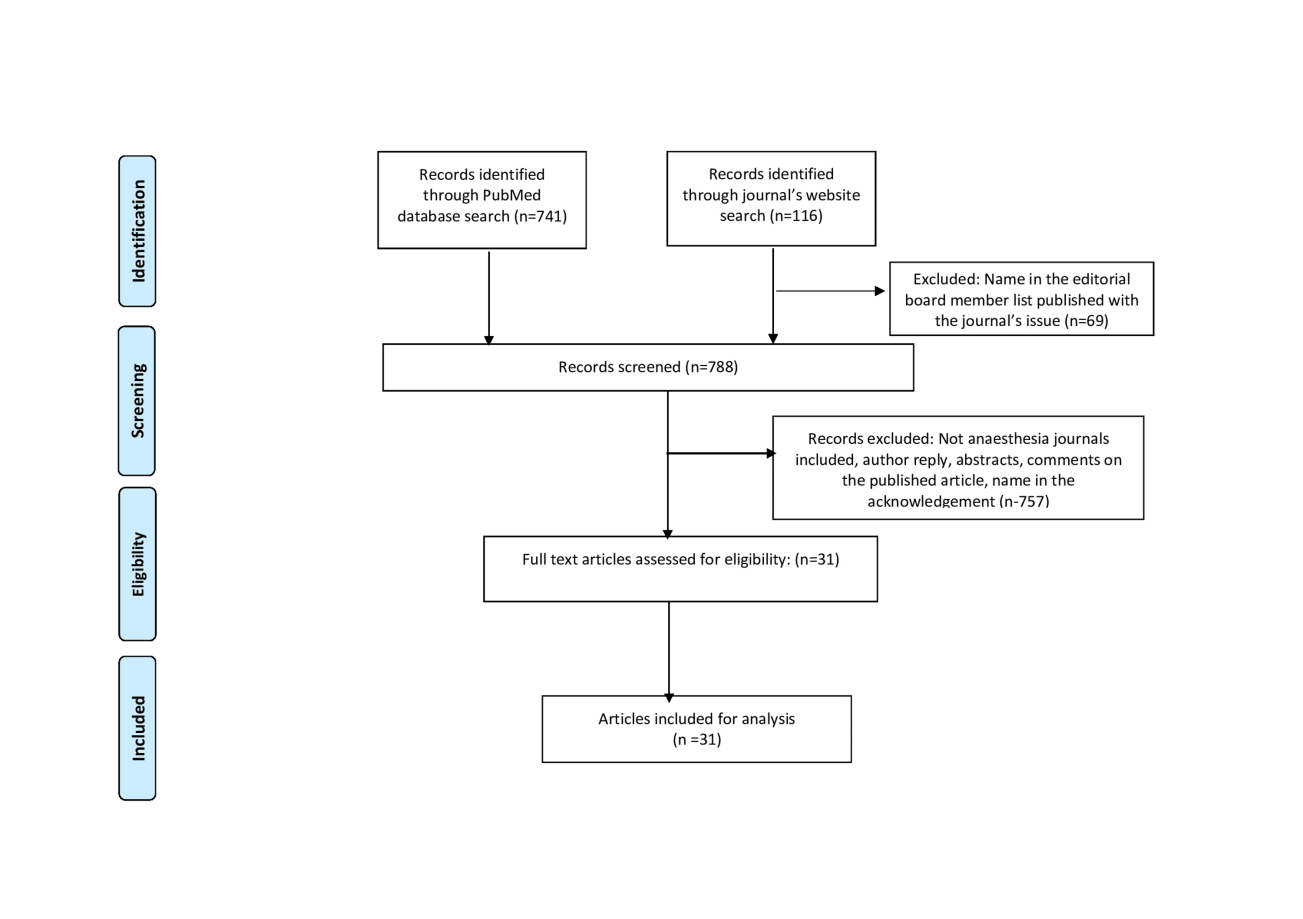
The Prisma flowchart The Prisma flowchart diagram summarizes the literature search

The Prisma flow chart diagram summarizes the literature search (Figure 1). Out of the 788 articles retrieved (Figure 1), the author found 31 publications in 10 journals from two of the seven emirates of the UAE. Table 1 highlights the journals included and a number of publications from the UAE. Table 2 shows the journals in which articles were published, the leading publication areas, contributing hospitals, and their locations.

**Table 1:** Journal and number of publications Impact factors for the journals and publications from the UAE. *The impact factor measures the average number of citations received in a particular year by papers published in the journal during the two preceding years. 2023 Journal Citation Reports (Clarivate Analytics, 2024).

Journal	Impact factor*	Number of publications from the UAE
British Journal of Anesthesia	9.1	5
Anesthesia	7.5	5
European Journal of Anesthesiology	4.2	5
Anesthesia, Critical Care and Pain Medicine	3.7	-
Anesthesiology	9.1	1
Anesthesia and Analgesia	4.6	1
Journal of Clinical Anesthesia	5.0	1
Regional Anesthesia and Pain Medicine	5.1	6
International Journal of Obstetric Anesthesia	2.6	1
Journal of Neurosurgical Anesthesiology	2.3	3
Journal of Cardiothoracic and Vascular Anesthesia	2.3	3
Pediatric Anesthesia	1.7	-

**Table 2:** Bibliometric analysis of publications from the UAE in anesthesia journals (2019-2023): area, author, and article types BJA: British journal of anesthesia, ACCPM: Anesthesia critical care pain medicine, EJA: European journal; of anesthesiology, anesthesia and analgesia, JCA: Journal of clinical anesthesia, RAPM: Regional anesthesia and pain medicine, JCVA: Journal of cardiothoracic and vascular anesthesia, JNSA: Journal of neurosurgical anesthesiology, IJOA: International J of obstetric anesthesia, Ped Anesth: Pediatric anesthesia, UAE: United Arab Emirates (UAE) CC: Cleveland clinic, HP: Health point, CH: Corniche hospital, TW: Tawam hospital, MC: Mediclinic city hospital, CMC: Clemenceau medical center, KC: King’s College a,b,c,d,e:  the same UAE author *-Co-authors from multiple international institutions

Journal		Type of publication	Abu Dhabi city			Al Ain City			Dubai city		
			Institute	Subject	UAE Author position (Total authors)	Institute	Subject	UAE Author position (Total authors)	Institute	Subject	UAE Author position (Total authors)
Europe	BJA	Editorial	CC^a^	Intensive care	1^st^ (2)*	—	—	—	—	—	—
		Original Res	CC^b^	Regional anesthesia	5^th^ (15)*	—	—	—	—	—	—
		Correspondence	CC^a^	Covid	1^st^, 2^nd^ (4)*	TW^c^	Medication error (ME)	1(1)	—	—	—
		Correspondence	HP	Medication	1 (1)	—	—	—			
	Anesthesia	Editorial	—	—	—	—	—		MCH^d^	Medicoleg-al (ML)	1^st^ (2)*
		Review	—	—	—	TW^c^	ME	1^st^ (3)*	MCH^d^	ML	1^st^ /(2)*
		Review	—	—	—	—	—	—	MCH^d^	ML	1^st^ (2)*
		Review	—	—	—	—	—	—	MCH^d^	ML	1^st^ (2)*
	EJA	Review	—	—	—	TW^c^	ME	1 (1)	—	—	—
		Review	—	—	—	TW^c^	ME	1(1)	—	—	—
		Guideline	CC^a^	PM	4^th^ (14)*	—	—	—	—	—	—
		Guideline	CC^a^	Vascular	1^st^ (13)*	—	—	—	—	—	—
		Guideline	CC^a^	Regional anesthesia	3^rd^ (13)*	—	—	—	—	—	—
USA	Anesthesiology	Images (Education)	CC	Pediatric	1 (3)*	—	—	—	—	—	—
	A&A	Special article	—	—	—	—	—	—	CMC	Patient safety	4^th^ (15)*
	JCA	Editorial	—	—	—	TW^c^	ME	1(1)	—	—	—
	RAPM	Original Res	CC^b^	Regional anesthesia	14^th^ (21)*	—	—	—	KC^e^	Regional anesthesia	9^th^ (10)*
		Original Res	CH^e^/CC^b^	Regional anesthesia	14^th^ and 54^th^ (92)*	—	—	—	KC^e^	Regional anesthesia	14^th^ (62)*
		Review	—	—	—	TW^c^	ME	1 (2) )*	—	—	—
		Guideline	CC^b^	Regional anesthesia	23^rd^ (42)*	—	—	—	—	—	—
Sub-specialty	IJOA	Correspondence	—	—	—	TW^c^	ME	3^rd^ (3)*	—	—	—
	JCVA	Review	—	—	—	TW^c^	ME	1(1)	Al Jalila	Cardiac	3^rd^ (9)
		Case report	—	—	—	—	—	—	Al Jalila	Cardiac	1^st^ (4)
	JNSA	Original Res	CC^a^	Neuro	7^th^ (16)*	—	—	—	—	—	—
		Original Res	CC^a^	Neuro	13^th^ (22)*	—	—	—	—	—	—
		Review	CC^a^	Neuro	1^st^ and 2^nd^ (4)*	—	—	—	—	—	—

Publication Analysis

More than two-thirds of the publications originated from the Abu Dhabi Emirate, with 45% from Abu Dhabi City and 25% from Al Ain City. Dubai City (Dubai Emirate) contributed 29% of the publications. The other five Emirates did not contribute to any publications. Table 2 provides a detailed breakdown of the journals included and the subject contributions in these journals. Notably, most publications were in American journals (55%), while 45% were published in three European journals (anesthesia, BJA, and EJA). Only five hospitals contributed to more than one publication during the five-year study period. Our analysis showed that there is less than one publication per year per million inhabitants in the UAE (Supplemental File). For the inhabitants of Abu Dhabi, Al Ain, and Dubai municipal areas, the yearly publication ratio was 1.5, 2, and 0.5 per million, respectively (Table 3). 

**Table 3:** Number of publications and population of the UAE’s large cities. (Population source: Wikipedia, 2023) Total population of the UAE is approximately 10 million.

Name	Emirate	Municipal population	Number of publications
Dubai	Dubai	3,564,931	9
Abu Dhabi	Abu Dhabi	1,807,000	14
Sharjah	Sharjah	1,405,000	-
Al Ain	Abu Dhabi	846,747	8
Ajman	Ajman	490,035	-
Ras Al Khaimah	Ras al Khaimah	191,753	-
Fujairah	Fujairah	118,933	-
Umm Al Quwain	Umm Al Quwain	59,098	-

Publication Focus

The main areas of publications were regional anesthesia (22.5%) and medication errors (25%; Table 2). The subspecialty publications were mainly for neurosurgical and cardiac anesthesia. Only one correspondence was published in the obstetric anesthesia specialty journal, while there was no publication in the pediatric anesthesia specialty journal. Notably, no publications on basic science or experimental (laboratory-based) studies indicated a significant opportunity for future research. 

Study Types

Nearly one-third of the articles published were review articles (29%), followed by original research (28%). UAE authors contributed to international guidelines (n=4) and had opportunities to write editorials on three occasions.

Authorship/International Collaboration

Nearly three-fourths (24 of 31) of the publications resulted from multinational authorship, highlighting the strong spirit of international collaboration in the field. It was particularly evident in 13 of 14 publications from the Abu Dhabi region, which were all multinational. All the original articles were international collaborations related to regional or neurosurgical anesthesia. When the publication involved only one or multiple authors working in the UAE (n = 8; 25.8%), it was predominantly from a single author in the Al Ain region. Overall, two authors (one from Abu Dhabi and one from the Al Ain region) contributed over 50% of the publications (eight for each of both authors) from the UAE (Table 2). From Abu Dhabi, Al Ain, and Dubai, the first author position was for six of 14, seven of eight, and five of nine articles, respectively. As corresponding authors affiliated in the UAE, six, seven, and two articles were published from Abu Dhabi, Al Ain, and Dubai.

Gender

One female author contributed to the research field with two original research articles and one guideline to the regional anesthesia and pain medicine journal. All three were multinational collaborative publications, and her author position was 14th (of a total of 21), 16th (of a total of 92), and 23rd (of a total of 42).

Discussion

Our study, to the best of our knowledge, is the first to quantitatively assess academic publications in high-impact mainstream and subspecialty journals by the anesthesia departments in the UAE. This unique contribution serves as a starting point, delving into the academic capacity and potential of the UAE, including the research conducted by departments and the involvement of anesthetists. We adopted a comprehensive approach to ensure that all relevant UAE anesthesiology contributions are considered and collected. The broad considerations allowed us to gain insights into the UAE's international collaborative research efforts in anesthesiology, emphasizing the importance of being part of a global community. This data underscores the significance of understanding the UAE's role in the worldwide research community and trends in clinical publication areas. 

The distribution of publications across the UAE is an interesting factor in understanding the academic landscape in anesthesiology. Our study demonstrates that publication activities are limited to a few Abu Dhabi and Dubai hospitals. Of the 32 publications included, 22 (69%) were from the two hospitals in the Abu Dhabi Emirate. Abu Dhabi has an area of 85% among all seven Emirates, with three large regions. The highest number of academic publications per million inhabitants was from Al Ain. One study examining international publishing trends in anesthesia over five years concluded that the geographic distribution of the publications on anesthesia must not only be analyzed in absolute numbers because, although the USA was the most productive by numbers, Finland, Sweden, and Denmark were the most productive countries per million inhabitants (8.8, 7.2, and 6 publications/year, respectively) [8]. Overall, there is a limited contribution to anesthesia and its subspecialties from the UAE. There is scope for initiating multicenter research among UAE hospitals.

The focus of the publication is a crucial aspect to consider. Although limited, publication topics demonstrate the depth and breadth of the articles published. Nearly one-third (31%) of the articles published were review articles, emphasizing synthesizing existing knowledge. Original research (28%) followed, indicating a commitment to advancing the field. All the original articles were international collaborations related to regional or neurosurgical anesthesia. UAE authors also contributed to international guidelines and wrote editorials, showcasing their thought leadership in the field. Future research should also investigate scholarly work for pain medicine and critical care specialty journals because some anesthesia departments may provide clinical services for these specialties.

The present investigation highlights only a few anesthetists published in high-impact anesthesia journals. For example, two authors (one from Abu Dhabi and one from the Al Ain region) contributed 50% of the publications (eight for each of both authors) from the UAE. When the publication originated within the UAE only (n=8; 25%), it was predominantly from a single author in the Al Ain region. This suggests that the UAE anesthesia community is predominantly providing clinical services. It may be because almost all anesthetists working in the UAE are recruited to provide clinical services, and most migrate for economic reasons. The significant gender disparity could be due to a predominantly higher number of male anesthetists working in the UAE. However, the author could not obtain data from any source for the total number of anesthetists working, their grades, or their gender.

The dearth of publication activities could be due to institutional infrastructure deficiencies (e.g., time, funding), anesthesia leadership (e.g., inexperience, unfocused), lack of recognition or rewards, no promotion pathway, and recruitment mainly for clinical work [9]. Several interrelated economic and non-economic factors may ensure and enhance excellence in the institution or university and the country. These factors include but are not limited to, gross domestic product/capital, research and development infrastructure and expenditure, local regulation promoting research, funding mechanisms for aspiring physician-scientists, and several experienced academic staff and mentors to support junior doctors and residents [10]. Gender representation in the field of anesthesia in the UAE is an area that requires attention. Some recent articles have addressed the problems that anesthesia departments can face and suggested solutions to support aspiring anesthesia physician-scientists [9,11]. Clinical service needs and academic performance-based compensation systems should be integrated in the UAE. It can be efficacious in a large anesthesia department [12].

The bibliometric analysis method examines how much the individual, regional, or specific group of countries, continents, or global contribution to the specialty of anesthesia is in terms of publications [5,6,13]. Unlike systematic reviews and meta-analyses, the method provides a feasible approach to summarizing the quantitative output of extensive data and does not require in-depth quality analysis [14]. Nevertheless, volume and quality matter; however, the problem lies in assessing the latter. The impact of the paper (or, as a surrogate, the impact factor of the journal in which the paper is published) is of some relevance [15] and provides an indirect indication. One recent review summarizes journal-based and article-based measures to identify impact [16].

Limitations

The results of the bibliometric analysis do not provide information about the clinical significance or outcome importance of the publications included. Our search included only the last five years, chosen as a snap period to identify the nature of publications and highlight trends. We included many articles, including those considered to have less evidence (e.g., editorials). It is also impossible to find the actual submission rate for included journals and, if submitted, the causes of rejection. In this review, journals are included for analysis only based on their impact factor; however, there are other commonly used journal-based quality metrics, e.g., cite score.

## Conclusions

Our investigation has revealed that the overall output of academic publications from the anesthesia departments in the UAE is currently low. The majority of publications were from two hospitals in the Abu Dhabi Emirate, and limited anesthetists are involved, with two contributing to more than 50% of publications. The reason may be that the anesthesia workforce provides clinical services, and academic work has no specific incentives. However, this also presents an opportunity for improvement. There is a need to integrate clinical and productivity-linked academic incentives to inspire and motivate the anesthesia community to improve research and its related contribution to anesthesia in the UAE. This could be a significant step towards enhancing academic contributions in the UAE's anesthesia field.
